# Contagion and Infection

**Published:** 1899-10

**Authors:** 


Contagion and Infection.—Grigg discusses the significance of these two terms, quoting various authorities as to their usage, and concludes that from his standpoint "an infectious disease is produced by a pathogenic micro-organism that lives and propagates in a suitable soil on the outside and independent of man, but when taken into the system and absorbed is capable of producing certain diseased conditions which are not directly transmissible from patient to host; while a contagious disease is produced by a pathogenic micro-organism that propagates in the afflicted patient and which, after escaping from said patient without undergoing further change and on being taken into the system of a prospective host and being absorbed, is capable of producing certain diseased conditions and is transmissible from patient to host, either directly by contact or indirectly as by fomites." He thinks it is of some importance to make this distinction clear.—*Journal American Medical Association.*

